# Designing animal-friendly behavioral tests for neuroscience research: The importance of an ethological approach

**DOI:** 10.3389/fnbeh.2022.1090248

**Published:** 2023-01-10

**Authors:** Raffaele d'Isa, Robert Gerlai

**Affiliations:** ^1^Institute of Experimental Neurology (INSPE), Division of Neuroscience (DNS), IRCCS San Raffaele Scientific Institute, Milan, Italy; ^2^Department of Psychology, University of Toronto Mississauga, Mississauga, ON, Canada

**Keywords:** behavioral testing, ethology, refinement, animal welfare, animal-friendly, rodents

Behavioral tests have three key elements: (1) a motivating factor (what motivates the animal in the test); (2) an observable behavior (which behaviors we may expect the animal exhibits in response to the test); (3) a measurable outcome (a quantifiable variable associated with the behavioral response).

For example, in the rodent step-through passive avoidance test (see d'Isa et al., 2014 for a brief history of the test), the animal is released into a strongly illuminated chamber connected to a dark zone. Being naturally photophobic and preferring dark areas, mice and rats will rapidly move from the well-illuminated zone into the dark zone, a behavior that in the wild is useful to avoid being seen by predators. When the animal enters the dark zone, it receives an electric shock. After the initial exposure to this apparatus (the training), the animal is released a second time in the same apparatus for a memory test, but this time without the shock deliverer being active. If the animal remembers receiving the shock, the dark zone should now be perceived as dangerous and hence avoided. In this test, the motivation is fear, the observable behavior is avoidance of a dangerous (dark) zone, and the quantifiable outcome is the latency to enter the dangerous zone, which thus serves as an index of memory. The longer is the latency, the stronger is the memory of the past electric shock exposure.

Behavioral testing of rodents in a laboratory setting started in the 1890's with the studies of Thomas Wesley Mills (1847–1915) from McGill University (Montreal, Canada) and of Linus Ward Kline (1866–1961) and Willard Stanton Small (1870–1943), both from Clark University (Worcester, Massachusetts, USA) (Mills, 1895, 1898; Kline, 1899a,b; Small, 1899, 1900, 1901). These studies were preceded by ethological, purely observational, studies on rodent behavior in the wild, e.g., Mills's studies on squirrel behavior (Mills, 1888, 1890, 1893), but it was only in the period 1895–1900 that behavioral tests for studying rodent behavior and psychology in a laboratory setting started to be designed. Mills also observed the behavior of two squirrels he captured and kept for few months, reporting, for example, how ethologically relevant behaviors could be observed, like nest-building and food storing, and how one of them learned to eat from his hand and enjoyed running on a running wheel that was installed in its home-cage (Mills, 1888). Although this was a first step for the study of rodent behavior in a controlled environment, the report with the findings was only anecdotal and appeared inserted within a paper on the behavior of squirrels in the wild. Two important elements of scientific testing were absent: systematic observations (i.e., observations at pre-set time-points according to a specific rationale) and choice of one or more quantitatively measurable behavioral outcomes as variables of interest. A systematic and quantitative study of rodent behavior in laboratory had yet to come.

Mills was the first, in the mid 1890's, to introduce for rodents the ontogenetic diary method, which consisted of observing and describing step by step the different developmental stages of a species, starting from the day of birth (Mills, 1895). Applying this method, he studied systematically the physical and psychological development of guinea pigs through a daily monitoring in a laboratory setting. In addition to purely observational studies of development, he also used some basic behavioral tests, e.g., reflex tests or taste reactivity tests, in which only qualitative responses were recorded (Mills, 1895, 1898). On the other hand, Kline and Small introduced behavioral tests aimed at specifically evaluating cognition. In 1898, a Clark University colleague of Kline and Small, Colin Campbell Stewart (1873–1944), published a study on the effects of alcohol, barometric pressure and type of diet on rat daily voluntary wheel running activity, as assessed by a revolving drum connected to automatic counters recording the total number of revolutions (Stewart, 1898). Stewart was the first to perform a quantitative rodent test of motor activity. The experiments of Stewart, from the Biology Faculty, inspired Kline, from the Psychology Faculty, the idea to choose rats as animal models for his research on learning processes (Kline, 1928; Miles, 1930). Kline designed a rat problem box (Kline, 1899a), while Small, who worked in the same laboratory as Kline, was the first to use a maze in the history of behavioral neuroscience (Small, 1901). In Kline's test, the task was finding the entrance of a box and retrieving the food contained inside it. Time to retrieve the food was recorded over multiple trials to assess learning. In Small's test, the task was finding food placed in the central zone of a complex maze inspired by the design of Hampton Court Maze, the well-known hedge labyrinth in England. Time to find the food and number of errors (entering a blind alley) were recorded over multiple trials. The ones performed by Kline and Small were the first quantitative rodent cognitive tests in the history of behavioral neuroscience. Regarding the motivating factor, both Kline and Small employed hungry rats in their cognitive tests, and food deprivation became a widely employed protocol in the subsequent studies using appetitive (reward-based) learning tasks.

However, since the dawn of behavioral neuroscience, researchers have been aware of the fact that hunger is only one possible motivator. Small himself wrote: “I trust the reader will not “jump” to the conclusion that no other motive would be workable. Hunger is merely the most fundamental” (Small, 1900). Noticing that well-fed rats still retrieved food during the task, he added: “Their performance of the task without the incitement of hunger can hardly be accounted for except upon the basis of a hoarding instinct almost as imperative as hunger” (Small, 1900). Nevertheless, these specifications seem to have been scarcely considered by the following researchers. Indeed, most behavioral tests designed up to the 1950's were based on rewards or punishments and commonly employed food deprivation or electric shocks, respectively.

Another classical avoidance task is, for example, the shuttle-box active avoidance, first conceived by Lucien Warner in the early 1930's (Warner, 1932), where instead of having to stay in the illuminated zone to avoid the shock, i.e., instead of not moving and being passive, the appropriate response is to actively move over to an opposite zone to avoid the shock when a stimulus (a tone or a light) announces its imminent release. In this task, the experimenter may have to employ a large number of shocks (even hundreds) over several days of training before animals reach high rates of shock avoidance and cognitively impaired animals could still show low rates of shock avoidance even at the end of the training (Montag-Sallaz and Montag, 2003; Cain, 2019). Painful stimulation, along with being ethically undesirable when dealing with any sentient organism, also leads to methodological complications for the experimenters. Indeed, pain generates stress, which is a major confounding factor in animal research. Still today, classical avoidance tests employ electric shocks, while many other traditional tests, although not utilizing painful stimuli, feature highly stressful conditions including starvation, water deprivation or pharmacologically induced sickness to motivate the animals to perform a task. However, an increasingly accepted view is that absence of pain and reduction of stress during behavioral testing are fundamental for both animal welfare and reproducibility of experimental results, and unless pain or stress is the main focus of the study, these conditions should be avoided as much as possible. In order to respond to such ethical and methodological concerns, several animal-friendly tests have been designed. However, since not all behavioral domains of investigation currently have such animal-friendly options, the development of new animal-friendly tests is an important goal for modern behavioral neuroscience.

## How to design an animal-friendly behavioral test

Compared to shuttle-box active avoidance, step-through passive avoidance, introduced in the 1960's (Kopp et al., 1966; Jarvik and Kopp, 1967), is considerably less stressful and in the test session no shock delivery is actually present. Nevertheless, the training session still features a brief painful stimulus. In an ideal animal-friendly behavioral test, the motivating factor should not be painful or stressful. Furthermore, the observable behavior should be natural (i.e., an ethologically relevant species-specific motor or postural pattern). Finally, the outcome variable associated with this behavioral response should be practical to measure in a laboratory setting through a method that is safe for the animals (e.g., direct observation, videorecording, videotracking, audiorecording of ultrasonic vocalizations, photocell actimetry, weight sensors, infrared thermometry and other non-invasive methods). These are the three main characteristics that a behavioral test should have to be qualified as animal-friendly. Let us first focus on the motivational aspect of behavioral tests, the first of the key components mentioned above.

Ethologists have been stressing the argument that motivating factors are species-specific (Gerlai and Clayton, 1999a; Gerlai, 2021). A stimulus that is appetitive (rewarding) or aversive (punishing) for one species, may be neutral, or may have the opposite reinforcing value to another. Even among closely related species, the rewarding value of a stimulus may be remarkably different. Among felids, for instance, tigers like to bath in water, while lions do not. Hence a swimming pool may be a reward for the former, but not for the latter species, as found, for example, by Allison Hedgecoth who provided a water pool to a lioness and a tigress living together in the same environment in the Noah's Ark Animal Sanctuary of Locust Grove, Georgia, United States (Harries et al., 2020). The main issue, however, in the behavioral neuroscience literature is that systematic analysis of what motivates animals used in laboratory settings is often lacking, or that ethology research often does not intersect with biomedical studies.

Considering, for example, laboratory rodents, the house mouse (*Mus musculus*) and the common rat (*Rattus norvegicus*) are the two most widely used species in biomedical research. Their employment is almost universal in translational research studying mechanisms of central nervous system disorders. Most studies that require aversive stimuli with rodents use electric shocks. But electric shocks are rather unnatural stimuli. However, almost no one considers what consequences may result from the unnatural aspect of this stimulus. It is just assumed that pain is pain, and that electric shock-induced pain is relevant and strongly motivating. Most scientists do not even consider what complication this electricity passively running through the body of the animal, including its brain, may cause with respect to neuronal activity: such electric currents may alter synaptic function and numerous underlying molecular mechanisms. Similarly, studies that employ appetitive stimuli, almost always use food that the experimenter picks out based on tradition, habit or just personal preference. Comparative analyses of what food types, food quantities, food textures and food sizes are most preferred by rats or mice are often not considered, or have not even been conducted. Briefly, as animals are, through evolutionary processes, adapted to their natural environment, they possess species-specific characteristics that represent genetic predispositions, instincts in colloquial terms, that determine, or at least heavily influence what they like, what they dislike, and how they respond to these stimuli. Taking these species-specific features into account is thus a must in animal-friendly experimental designing (Gerlai and Clayton, 1999a,b). In rodents, a typical animal-friendly motivating factor is neophilia (attraction for novelty), which drives, for instance, object exploration behavior in the object recognition test (d'Isa et al., 2014), head-dipping in the hole-board test (d'Isa et al., 2021a) and arm alternation in the spontaneous alternation T-maze (d'Isa et al., 2021b). A similar example is the continuous spontaneous alternation test using a T-maze, which utilizes novel place preference to study short-term spatial memory in rodents (Gerlai, 1998).

Regarding the second key element mentioned above, the observable behavior in an animal-friendly test should be naturally displayed by the animal (e.g., should be part of the ethogram). Preferably, it should be a spontaneous behavior, an instinctive response that requires no pre-training, during which typically punishments and rewards are used by the experimenter to lead to a target behavior. Punishments are commonly painful stimuli (as electric shocks), while rewards, as food or liquids, are often associated with food-deprivation or water-deprivation, in order to use hunger or thirst as motivating factors. Lack of the need for pre-training makes the test more animal-friendly because it avoids punishments and deprivations, and is also time-saving for the experimenter. It is, however, also possible to use conditioned behaviors in an animal-friendly way, if certain conditions are respected. In particular, rewards should not be associated with a previous aversive state. Chow and colleagues, for instance, designed a reward-based cognitive test for gray squirrels in which no food-deprivation or water-deprivation was employed (Chow et al., 2017). The motivation of the rodents was ensured simply by using food rewards (hazelnuts) that were different from their daily diet (seeds, fresh fruit and vegetables), i.e., novelty alone was sufficient to motivate the animals. Novelty-seeking and exploratory drive (i.e., the motivation to learn about new places and/or new inanimate or animate components of the environment) are almost universal among animal species, and certainly have been shown for laboratory rodents (Gerlai et al., 1990; Crusio, 2001). In fact, stabilizing natural selection has been inferred for exploratory behaviors from fish to mammals, as it leads to an optimal level of activity ensuring the ability of the animal to find resources, including food, water and mates, as well as escape routes leading away from predators (Gerlai et al., 1990; Crusio, 2001). The use of novelty as a motivator may not be appropriate in some research contexts and, for certain studies, aversive stimulation may be required. However, even in such cases, painful punishments could be and should be substituted with non-painful aversive alternatives, for example, air-puffs. Indeed, air-puffs have been efficiently employed to elicit robust conditioned place avoidance negating the need for using any painful stimuli (d'Isa et al., 2011). Even for studies specifically focused on fear reactions, alternatives to painful stimulation are available. Odor of predators (e.g., fox's urine, or an extract from it) has been efficiently used to induce avoidance reactions and fear without previous painful stimulation (Blanchard et al., 2003).

The main steps for designing an animal-friendly test can be summarized as follows: (a) prepare a list of behaviors typical of the species (the ethogram), along with what stimuli may induce these behaviors, i.e., the motivating forces; (b) exclude behaviors induced by pain, physical suffering or psychological stress; (c) from the remaining, choose a behavior that can be studied through an apparatus that can be used in a laboratory setting; (d) choose which outcomes could be measured, safely for the animals, in the most efficient and precise way in order to provide quantitative experimental data. Let us examine an experimental example of how these steps may be accomplished.

A typical behavior of rodents is food hoarding, that is, collecting and hiding food as supply storage for times of food scarcity. This behavior can be observed in more than 180 rodents (Zhang et al., 2022). This is an adaptive behavior that is observable both in nature and in the laboratory setting. It is an instinctive behavior that does not require pre-training. Two main strategies are adopted by food hoarding rodents. Scatter hoarders, as gray squirrels, hide food in many dispersed small hoards. On the other hand, larder hoarders, as hamsters, store food in one large hoard, named the larder. A classification of the hoarding strategies of 183 rodents is provided by Zhang and colleagues (Zhang et al., 2022). These hoarding behaviors may be utilized by the experimenter to devise behavioral tests of motivation (during the food accumulation phase) or of spatial memory (during the subsequent phase of food retrieval from the spatially separated hoards). For motivation tests, easier to study in larder hoarding rodents, the measurable outcome could be the total weight of the seeds or pellets collected and stored in a fixed amount of time. For spatial memory tests, which would be best studied with scatter hoarding rodents, the recorded outcome could be the number of errors in finding the hoarding sites containing the previously stored food. Alternatively, spatial memory could be studied also in larder hoarders if, during the accumulation phase, the sources of food are multiple. Number of errors (returning to an already depleted food site) would serve as memory index. An apparatus for the testing of food hoarding behavior in a laboratory setting has been realized, for example, by Robert Deacon at Oxford University (Deacon, 2006).

An ethological approach may be useful to devise animal-friendly behavioral tests for two reasons. On the one hand, it may help researchers to choose among the elements of the ethogram a behavior that does not require painful or stressful motivating factors. On the other hand, among a taxonomical family of species (for example rodents), it may help researchers to select the most suitable species for a certain test. Let us return to the example we mentioned above. Laboratory mice are larder hoarders, just like hamsters, but their propensity to hoard is relatively low under baseline conditions. In order to avoid food-deprivation, long testing sessions may be required to obtain replicable results, including, e.g., overnight testing sessions (Deacon, 2006). Hamsters, on the other hand, have a high propensity to hoard (Vander Wall, 1990; Harris, 2017). Up to 90 kg of food have been found in hamster burrows (Nowak and Paradiso, 1983). Among food hoarders, they display a specific behavior known as cheek pouching, that is accumulating food in cheek pouches, specialized pockets that allow food transportation. Instead of eating the food items, hamsters keep the food items in their mouth to carry them to a safe place for storage (the larder). Importantly, hamsters easily show this behavior even when they are not hungry, with a latency to hoard within 2 min (Montoya and Gutiérrez, 2016). This peculiarity of hamsters makes them particularly suitable as animal models for scientists who want to design an animal-friendly reward-based memory test that does not require any previous starvation.

Another rodent, the chinchilla (*Chinchilla lanigera*), displays a peculiar behavior known as sand-bathing: when presented with a box full of sand, it will readily start rolling in the box, rotating along its longitudinal axis, to rub its fur in the sand (Stern and Merari, 1969). This natural and spontaneous behavior can be easily elicited in a laboratory setting and sand could be used as an animal-friendly reward in instrumental learning tests without the need of any previous deprivation condition (Redman, 1974).

Eastern woodrats (*Neotoma floridana*), also known as pack rats, have a special attraction for shiny objects, which they readily approach, pick up and bring to their nest, where they collect them (Bradley et al., 2022). This natural tendency of woodrats could be used in behavioral tests, employing small metal objects, as stripes or balls of aluminum foil, as motivators (Kaufman and Kaufman, 1984).

Of course, the issue is that quite often neurobiological, genetic, or other methods may not be as readily available, or as sophisticated, for such species as hamsters and chinchillas as for the favorites of biomedical research, mice and rats. How can we solve this conundrum? Firstly, we could improve biotechnological methods for the so called “alternative” species. Secondly, we could improve our understanding of the ethology, the natural species-specific behavioral characteristics, of the preferred model organisms, e.g., of mice and rats. Certainly, advances in both of these areas have been made during the past few decades. Regarding the first area, numerous novel techniques may now be equally useable with mice and hamsters (and many other species). The CRISPR/Cas technology is a clear example (Kampmann, 2020). Concerning the second area, there have been research efforts adopting ethological approaches in mouse neurobehavioral genetics, as for instance testing mouse mutants in the wild (Dell'Omo et al., 2000; Vyssotski et al., 2002) or in laboratory environments more closely resembling a natural habitat, like Eco-HAB (Puścian et al., 2016; Winiarski et al., 2022). Anders Ågmo's research group at University of Tromsø recreated a seminatural environment in the laboratory for the evaluation of rat behavior (Chu and Ågmo, 2014), a method that has been employed in several subsequent studies (Chu et al., 2015; Houwing et al., 2019; Le Moëne et al., 2020; Heinla et al., 2021). In the testing sessions, which may last days, rat behavior is continuously video-recorded and subsequently scored off-line by the researchers. Notably, another important ethological approach of the new century is testing the animals not in a setting designated uniquely for testing sessions, but rather in the permanent housing environment in which they commonly live (Mingrone et al., 2020; Voikar and Gaburro, 2020). In nature, most rodents build burrows (or occupy pre-existing holes or burrows) to use them as homes (long-term inhabiting spaces), in which they return to sleep, store food, seek shelter from the elements, keep warm, hide from predators, give birth, raise the pups and share a social life with conspecifics. Rodents develop a strong bond with their home and show a territorial behavior toward it, actively defending it from possible invaders. In the laboratory, if an unfamiliar conspecific is placed in the home-cage of mice or rats, the intruder will rapidly be attacked by the resident animal (Koolhaas et al., 2013; Ruzza et al., 2015). The home-cage is the place where laboratory rodents feel safest and where they are more likely to display spontaneous natural behaviors. Thus, the idea of testing in the home-cage has been gaining considerable attention in the past few years, and several home-cage automated multi-variable recording systems have been developed, e.g., the IntelliCage (Galsworthy et al., 2005; Kiryk et al., 2020; Iman et al., 2021), PhenoMaster (Urbach et al., 2008; König et al., 2020), Actual-HCA (Bains et al., 2016; Mitchell et al., 2020) and SmartKage (Ho et al., 2022). Automated home-cage testing systems have several advantages: (a) they allow behavioral phenotyping without human interference and without the consequent handling-related stress; (b) the animals are not tested in an external apparatus but in their familiar and well-known housing environment, which eliminates confounds arising from anxiety; (c) data collection is not restricted to a specific moment of the day, but can be performed continuously, 24 h a day, 7 days a week, allowing a more precise and realistic assessment of behavior; d) long longitudinal studies (lasting weeks, months or years), or even life-long studies, can be performed on the same animals with a continuous behavioral assessment, which is particularly relevant for developmental neuroscience and aging neuroscience; (e) interactive elements (e.g., levers, nose-poking ports, motorized doors and running wheels) may be installed in these home-cages, allowing not only detailed motor assessment, but also complex cognitive testing; (f) animals are tested in a natural social context while living together with other conspecifics, thus providing motor and cognitive measurements with a higher ethological validity and allowing additionally to monitor and analyze complex social interactions. Some of these automated home-cage testing systems are modular, allowing the connection of multiple cages to create a more complex envinronment. For instance, IntelliCage can be connected to two social boxes containing different social stimuli (Mitjans et al., 2017), while in ColonyRack mice can freely roam across 70 cages, arranged in a two-sided rack with fivs columns and seven rows, in which the cages are connected both horizontally and vertically (Zocher et al., 2020; Kempermann et al., 2022). The most recent innovation within this automated behavioral testing approach is connecting home-cages to mazes (Mei et al., 2020; Kohler et al., 2022), granting the experimental subjects free access to the novel test environment. This allows the animals to decide voluntarily when and for how long they explore the maze, similarly to what would happen in nature when rodents decide to leave their burrow for external exploratory excursions.

We believe that bringing closer the fields of ethology and neurobehavioral genetics or behavioral neuroscience will be the solution and will lead to cross-fertilization of these fields. Similarly to how the application of neuroscience-related knowledge to ethology led to the birth of neuroethology (i.e., the study of the neural basis of natural behaviors), the reverse could lead to an ethologically based neuroscience, or ethological neuroscience, which can be defined as the employment of knowledge of the natural behavior of animals in the wild to develop animal models of behavior and behavioral tests for neuroscience research. This ethologically based neuroscience can lead to animal-friendly testing approaches that will not only be more oriented toward the welfare of the animals involved, but also will provide more reliable and more replicable results for the experimenters.

## Concluding remarks: Reproducibility, replicability and refinement

Reproducibility is when we obtain the same results repeatedly by using identical methods (Kafkafi et al., 2018; Gerlai, 2019), whereas replicability is when we reach similar conclusions by adopting different methodologies (Kafkafi et al., 2018; Gerlai, 2019). Minimizing stress of the tested animals is a value in itself from an ethical point of view. However, since stress is a confounding factor that increases variability of experimental outcomes, minimizing stress is also fundamental to achieve methodologically sound scientific research. Why does research that ignores species-specific features lead to increased variability? Why is stress a confounding factor that reduces reproducibility? These are intriguing questions that would deserve specific research. The answer may lay in the fact that stress causes activation of the hypothalamic–pituitary–adrenal (HPA) axis, which in turn alters physiological processes regulating cognition and behavior (Moreira et al., 2016). HPA reactivity depends on genetic, epigenetic and environmental factors (Holmes et al., 2005), which makes it more difficult to predict than instinctive responses. Let us make some overarching theoretical points. Most animal research includes human handling. Human handling is extremely difficult to standardize (Crabbe et al., 1999). Even if handling was perfectly standardized, stress reactivity of the animals would not. Animals experiencing more stress due to the experimental procedures will be more responsive to human handling, which then will lead to elevated error variation in the behavioral test. In order to maximize experiment reproducibility, the best option is to minimize handling-related stress (Gouveia and Hurst, 2017). Considering, for instance, mice, although tail picking is the most commonly employed method of handling (Ueno et al., 2020), this method features tail lifting, tail suspension and swinging the animal over a void, which are highly stressful for the mice. Indeed, tail lifting, compared with alternative handling methods that do not require tail lifting, increases anxiety in the open-field test (Gouveia and Hurst, 2019) and elevated plus maze (Hurst and West, 2010), and it has been shown to reduce exploratory activity (Gouveia and Hurst, 2017), to increase aversion for the human handler in voluntary interaction test (Hurst and West, 2010) and to impair responsiveness to sucrose reward, indicating a reduction of reward's hedonic value (Clarkson et al., 2018). Several animal-friendly approaches are now available to avoid the negative impact of human handling on mice: (a) adopting non-aversive manual handling techniques, as open-hand retrieval through the cupping method (Hurst and West, 2010; Gouveia and Hurst, 2017, 2019; d'Isa et al., 2021b; Davies et al., 2022); (b) employing a tool to handle the mice, as a plastic handling tunnel (Hurst and West, 2010; Gouveia and Hurst, 2013, 2017, 2019; Sensini et al., 2020; Davies et al., 2022); (c) using automated home-cage testing systems in which behavioral outcomes are recorded without physical interaction with the human experimenter (Kiryk et al., 2020; König et al., 2020; Mitchell et al., 2020; Ho et al., 2022; Kohler et al., 2022; Winiarski et al., 2022).

Furthermore, not knowing the species-specific characteristics of the studied organism, for example, applying inappropriate motivators, forcing the animal to exhibit behavioral responses it would not normally perform, and measuring the behavior under artificial conditions that do not have much to do with the natural environment in which the animal evolved, all can elevate random error, simply because the individuals tested this way may have to find unique solutions to the problems, considerably increasing individual differences in the study (Gerlai and Clayton, 1999a,b). To put it in the words of the aforementioned pioneer of experimental behavioral research Willlard Stanton Small, “the experiments must conform to the psycho-biological character of an animal if sane results are to be obtained” (Small, 1901).

Animal-friendly tests utilizing species-specific features of the studied organism may not be always available or applicable, but, when they are, they should be employed as a first option, in order to maximize both animal welfare and repeatability of experimental results. When fully animal-friendly tests are not available, then the least stressful available test should be employed. In Table 1 we present a rating scale for behavioral tests based on their impact on animal welfare. This rating is not meant to be final, but rather a starting point to stimulate reflection and discussion on the differential stress impact of behavioral tests. We hope that in future an increasing number of studies will employ tests of class A (animal-friendly) and B (minimally stressful) and that, in accordance with a progressive refinement principle, new animal-friendly tests will be designed to substitute the more stressful alternatives.

**Table 1:** Rating of the impact of behavioral tests on animal welfare.

**Welfare rating**	**Features**	**Behavioral tests**	**References**
A	• Spontaneous behaviors • Conditioning only through rewards not associated with previous aversive situations or through non-painful disincentives • No food deprivation • No water deprivation • No forced water immersion • No painful stimuli • No distressful conditions	Novel object recognition	d'Isa et al., 2014
		Object location test	Murai et al., 2007
		Object exploration test	Steinbach et al., 2016
		Spontaneous alternation T-maze	d'Isa et al., 2021b
		Continuous alternation Y-maze	Detrait et al., 2010
		Continuous alternation T-maze	Gerlai, 1998
		Spontaneous 8-arm radial maze (no food deprivation, unbaited)	Haga, 1995
		Spontaneous 6-arm radial maze (no food deprivation, unbaited)	Alessandri et al., 1994; Opitz et al., 1997
		Spontaneous Dashiell hexagonal maze (no food deprivation, unbaited)	Giménez-Llort et al., 2007
		Free access rewarded 8-arm radial maze (no food deprivation, baited)	Mei et al., 2020; Kohler et al., 2022
		Hole-board test	d'Isa et al., 2021a
		Locomotor activity test	Visigalli et al., 2010
		Open-field test (in dim light)	McReynolds et al., 1967; Võikar and Stanford, 2023
		Emergence test	Paré et al., 2001
		Sociability test in the three-chambered apparatus	Gu et al., 2022
		Social vs. object preference test in the three-chambered apparatus	Lammert et al., 2018
		Social novelty preference test in the three-chambered apparatus	Kaidanovich-Beilin et al., 2011
		Social recognition test	Jacobs et al., 2016
		Opposite-sex partner preference test in the satellite cages apparatus	Linnenbrink and von Merten, 2017
		Mate choice test in the three-chambered apparatus	Nomoto et al., 2018; Guarraci and Frohardt, 2019
		Paced mating sexual behavior test	Zipse et al., 2000; Nedergaard et al., 2004
		Paced mating-induced conditioned place preference	Paredes and Alonso, 1997; Camacho et al., 2009
		Two-bottle taste preference test	Gaillard and Stratford, 2016; Strekalova, 2023
		Saccharin consumption test	Inostroza et al., 2012
		Voluntary wheel running	Goh and Ladiges, 2015
		Successive alleys test	Deacon, 2013a
		Nest-building test	Neely et al., 2019; Dorninger et al., 2020
		Burrowing test	Deacon, 2009
		Food hoarding test (without food deprivation)	Deacon, 2006
		Marble burying test	Angoa-Pérez et al., 2013; Witkin and Smith, 2023
		CatWalk gait analysis	Crowley et al., 2018; Pitzer et al., 2021
		IntelliCage automated home-cage testing	Vannoni et al., 2014; Kiryk et al., 2020
		SmartKage automated home-cage testing	Ho et al., 2022
		Eco-HAB automated home-cage testing	Puścian et al., 2016; Winiarski et al., 2022
		PhenoMaster automated home-cage testing	Robinson et al., 2013; König et al., 2020
		Actual-HCA automated home-cage testing	Bains et al., 2018; Mitchell et al., 2020
B	• Low psychological stress • No food deprivation • No water deprivation • No forced water immersion • No painful stimuli	Barnes maze	Rosenfeld and Ferguson, 2014
		Open-field test (in bright light)	Seibenhener and Wooten, 2015
		Light-dark transition test	Takao and Miyakawa, 2006
		Elevated plus maze	Walf and Frye, 2007
		Rotarod	Papale et al., 2017
		Pole test	Zhu et al., 2017
		Beam walking test	Luong et al., 2011
		Pup retrieval test	Lee et al., 2021; Winters et al., 2022
		Pup-rewarded auditory learning test	Besosa et al., 2020
C	• Moderate psychological stress • No food deprivation • No water deprivation • No painful stimuli	Morris water maze	d'Isa et al., 2011
		Grip strength test	Mandillo et al., 2008
		Inverted screen test	Deacon, 2013b
		Prepulse inhibition test	Valsamis and Schmid, 2011; Ioannidou et al., 2018
		Visual threat flight test	Huang et al., 2020; Yang et al., 2020
D	• Brief food deprivation (up to 18 h) • Brief water deprivation (up to 9 h) • No painful stimuli	Socially transmitted food preference test	Wrenn et al., 2003; Plucinska et al., 2012
		Social vs. food preference test	Reppucci and Veenema, 2020
		Hyponeophagia test	Deacon, 2011
		Water-rewarded social cooperation test	Feng et al., 2021; Shin and Ko, 2021
E	• High psychological stress • No food deprivation • No water deprivation • No painful stimuli	Forced swimming test	Castagné et al., 2011
		Tail suspension test	Can et al., 2012
		Predator odor test	Otsuka, 2017
		Counter-current swimming test	Matsumoto et al., 1996; Mizunoya et al., 2002
F	• Brief painful stimulation ( ≤ 2 s)	Passive avoidance test	Papale et al., 2017
		Single-shock fear conditioning	Poulos et al., 2016
		Shock-probe defensive burying test	Fucich and Morilak, 2018
G	• Repeated (2–5 events) or extended (>2 and ≤ 10 total s) painful stimulation	Multiple-shock fear conditioning	Shoji et al., 2014; Müller and Fendt, 2023
		Multiple-shock passive avoidance	Takahashi et al., 2018
		Tail-flick test	Chidiac et al., 2021
		Hot plate test	Lee et al., 2018
H	• Prolonged food restriction (from 18 h to weeks) • Prolonged water restriction (from 9 h to weeks)	8-arm radial maze	Crusio and Schwegler, 2005
		Cross maze	Pittenger et al., 2006
		Food-rewarded 5-choice serial reaction time task	Asinof and Paine, 2014
		Novelty-suppressed feeding test	Fukumoto and Chaki, 2015
		Water-rewarded 5-choice serial reaction time task	Birtalan et al., 2020
		Water-rewarded auditory decision making test	Jaramillo and Zador, 2014
		Water-rewarded labyrinth	Rosenberg et al., 2021
I	• Sickness induction	Lithium chloride-induced conditioned taste aversion	Lavi et al., 2018
		Lithium cloride-induced conditioned place aversion	Frisch et al., 1995
J	• Repeated (>5 events) or extended (>10 total s) painful stimulation	Shuttle-box active avoidance	Montag-Sallaz and Montag, 2003
		Learned helplessness test	Vollmayr and Henn, 2001; Silveira and Joca, 2023
		Formalin test	Teng and Abbott, 1998
K	• Potential physical injury (mild to severe)	Resident-intruder aggression test	Koolhaas et al., 2013
		Resident-intruder violence test	Haller et al., 2006; Koolhaas et al., 2013
L	• Lethal tests	Terminal sleep deprivation	Everson et al., 1989
		Drowning test	Richter, 1957

A suggested rating scale for behavioral tests in order of severity (from A to L) of the impact on the wellbeing and welfare of laboratory rodents is presented. For each rating class, features are described and a list of example behavioral tests is provided. The scale comprises twelve welfare classes, from A (no negative impact on animal welfare) to L (most severe negative impact). Important notes:

1. Test impact on animal welfare is species-specific. The suggested rating is specific for crepuscular/nocturnal rodents (as mice, rats and hamsters). For different species of rodents, the ratings may differ. For instance, for the degu (*Octodon degus*), a diurnal rodent species in which adults show strong preference for the lit compartment in the light-dark transition test (Popović et al., 2009), the Barnes maze would have a rating of A instead of B.

2. This classification of behavioral tests is based on the normal responses expected from wild-type or untreated/unmanipulated control rodents. However, as test-associated stress always derives from the interaction between the test and the experimental manipulations (e.g., mutations induced or drug treatment employed), even behavioral tests that are minimally stressful for untreated wild-types may lead to high distress in manipulated animals. Thus, the experimenter must always consider not only what wild-type control animals will do in the test, but also closely monitor how the mutant/treated animals respond in pilot studies, and revise the experimental protocol or choose alternative tests accordingly.

3. This scale is ordinal, but not linear. We are not assuming equal distances between classes.

4. Due to the degree of suffering inflicted on the animals, the tests reported in category J are considered unethical according to current ethical standards, and nowadays would not be approved by the institutional animal care and use committees (IACUCs), nor would receive legal authorization, in most countries performing scientific research.

5. The rating scale is only a suggested scale, a non-comprehensive working document that is meant to be debated, updated and expanded by the rodent research community.

## Author contributions

Rd'I and RG: conceptualization, writing, revision, and final approval of the manuscript. Both authors provided funding. Both authors contributed to the article and approved the submitted version.
